# Supplementary material for: Estimating the early impact of vaccination against COVID-19 on deaths among elderly people in Brazil: Analyses of routinely-collected data on vaccine coverage and mortality
Source: eClinicalMedicine. 2021 Jul 16;38:101036. doi: 10.1016/j.eclinm.2021.101036 (PMC8283303; doi:10.1016/j.eclinm.2021.101036)
Supplement: Supplementary file 1 [file mmc1.docx]

# ­­­­­

# **Captions for supplementary materials**

# Estimating the early impact of vaccination against COVID-19 on deaths among elderly people in Brazil:

# analyses of routinely-collected data on vaccine coverage and mortality.

**Table and Figure Captions:**

Supplementary table 1. Absolute number of deaths due to COVID-19 and to all other causes by epidemiological week according to age groups. Brazil,2021.

Supplementary figure 1. Proportionate mortality of individuals aged 80+ years due to COVID-19 relative to deaths at all ages due to COVID-19 by region and epidemiological week. Brazil, January to April 2021.

Supplementary figure 2. Proportionate mortality due to COVID-19 of individuals aged 70-79 and 80+ years relative to deaths at all ages due to COVID-19 by month. Brazil, May 2020 to May 2021.

Supplementary figure 3. Sex-specific proportionate mortality due to COVID-19 of individuals aged 70-79 and 80+ years relative to deaths at all ages due to COVID-19 by month. Brazil, May 2020 to May 2021.
