# Supplementary material for: Estimating the early impact of vaccination against COVID-19 on deaths among elderly people in Brazil: Analyses of routinely-collected data on vaccine coverage and mortality
Source: eClinicalMedicine. 2021 Jul 16;38:101036. doi: 10.1016/j.eclinm.2021.101036 (PMC8283303; doi:10.1016/j.eclinm.2021.101036)
Supplement: Supplementary file 2 [file mmc2.docx]

# ­­­­­

# **Supplementary materials**

# Estimating the early impact of vaccination against COVID-19 on deaths among elderly people in Brazil:

# analyses of routinely-collected data on vaccine coverage and mortality.

**Supplementary table 1. Absolute number of deaths due to COVID-19 and to all other causes by epidemiological week (starting January 3, 2021) according to age groups. Brazil,2021.**

| Age (years) | 0-9 | | 10-19 | | 20-29 | | 30-39 | | 40-49 | | 50-59 | | 60-69 | | 70-79 | | 80-89 | | 90+ | |
| --- | --- | --- | --- | --- | --- | --- | --- | --- | --- | --- | --- | --- | --- | --- | --- | --- | --- | --- | --- | --- |
| Cause | Other | COVID | Other | COVID | Other | COVID | Other | COVID | Other | COVID | Other | COVID | Other | COVID | Other | COVID | Other | COVID | Other | COVID |
| Week |  |  |  |  |  |  |  |  |  |  |  |  |  |  |  |  |  |  |  |  |
| 1 | 783 | 17 | 386 | 8 | 967 | 52 | 1,216 | 164 | 1,766 | 389 | 3,079 | 757 | 4,608 | 1,621 | 5,310 | 1,880 | 5,102 | 1,433 | 2,443 | 465 |
| 2 | 734 | 14 | 384 | 11 | 1,046 | 45 | 1,269 | 193 | 1,922 | 448 | 3,123 | 951 | 4,729 | 1,668 | 5,288 | 1,979 | 5,131 | 1,566 | 2,387 | 511 |
| 3 | 718 | 20 | 334 | 15 | 957 | 58 | 1,192 | 242 | 1,814 | 450 | 3,080 | 946 | 4,612 | 1,754 | 5,220 | 2,168 | 4,971 | 1,612 | 2,332 | 527 |
| 4 | 706 | 8 | 383 | 14 | 964 | 64 | 1,208 | 229 | 1,809 | 471 | 3,051 | 959 | 4,544 | 1,714 | 5,440 | 2,082 | 5,031 | 1,626 | 2,447 | 521 |
| 5 | 713 | 17 | 347 | 10 | 1,019 | 64 | 1,222 | 190 | 1,827 | 482 | 3,048 | 953 | 4,715 | 1,730 | 5,409 | 1,876 | 5,215 | 1,537 | 2,610 | 508 |
| 6 | 739 | 13 | 340 | 11 | 919 | 68 | 1,143 | 193 | 1,768 | 489 | 2,970 | 915 | 4,459 | 1,604 | 5,320 | 1,784 | 4,970 | 1,421 | 2,336 | 440 |
| 7 | 732 | 15 | 338 | 8 | 913 | 69 | 1,145 | 247 | 1,782 | 546 | 3,009 | 991 | 4,485 | 1,670 | 5,203 | 1,841 | 4,899 | 1,369 | 2,335 | 490 |
| 8 | 757 | 17 | 368 | 21 | 926 | 91 | 1,151 | 319 | 1,778 | 720 | 3,051 | 1,148 | 4,550 | 2,041 | 5,266 | 2,228 | 5,009 | 1,650 | 2,436 | 494 |
| 9 | 739 | 21 | 310 | 22 | 907 | 134 | 1,168 | 467 | 1,869 | 975 | 3,100 | 1,660 | 4,613 | 2,814 | 5,184 | 2,831 | 5,137 | 2,161 | 2,365 | 681 |
| 10 | 761 | 15 | 322 | 21 | 935 | 178 | 1,133 | 663 | 1,850 | 1,366 | 3,181 | 2,313 | 4,683 | 3,662 | 5,458 | 4,054 | 5,281 | 2,586 | 2,445 | 749 |
| 11 | 766 | 25 | 334 | 28 | 909 | 188 | 1,223 | 916 | 1,879 | 1,856 | 3,098 | 3,178 | 4,927 | 4,945 | 5,702 | 5,007 | 5,396 | 3,212 | 2,393 | 876 |
| 12 | 684 | 19 | 347 | 31 | 946 | 238 | 1,205 | 1,019 | 1,791 | 2,223 | 3,189 | 3,812 | 4,936 | 5,934 | 5,640 | 5,798 | 5,127 | 3,384 | 2,389 | 764 |
| 13 | 639 | 25 | 324 | 26 | 866 | 266 | 1,152 | 1,029 | 1,873 | 2,323 | 3,023 | 3,932 | 4,615 | 6,147 | 5,506 | 5,879 | 4,865 | 2,818 | 2,262 | 680 |
| 14 | 641 | 17 | 308 | 27 | 835 | 243 | 1,097 | 960 | 1,777 | 2,064 | 2,780 | 3,718 | 4,399 | 5,573 | 5,041 | 5,156 | 4,421 | 2,306 | 2,134 | 539 |
| 15 | 576 | 19 | 264 | 19 | 790 | 202 | 1,101 | 848 | 1,680 | 1,823 | 2,719 | 3,256 | 4,313 | 4,871 | 4,895 | 4,387 | 4,273 | 1,809 | 1,966 | 470 |
| 16 | 641 | 23 | 283 | 16 | 746 | 221 | 994 | 719 | 1,678 | 1,591 | 2,645 | 2,685 | 4,077 | 4,262 | 4,590 | 3,507 | 4,136 | 1,424 | 1,983 | 413 |
| 17 | 551 | 11 | 256 | 22 | 710 | 171 | 891 | 643 | 1,361 | 1,414 | 2,319 | 2,511 | 3,567 | 3,725 | 3,995 | 2,654 | 3,553 | 1,188 | 1,697 | 311 |
| 18 | 445 | 11 | 181 | 17 | 516 | 122 | 708 | 565 | 1,148 | 1,217 | 1,897 | 2,135 | 2,800 | 3,122 | 3,155 | 1,923 | 2,963 | 917 | 1,414 | 278 |
| 19 | 326 | 9 | 149 | 20 | 381 | 117 | 572 | 473 | 934 | 1,146 | 1,567 | 1,836 | 2,407 | 2,393 | 2,645 | 1,367 | 2,595 | 787 | 1,176 | 258 |

**Supplementary figure 1. Proportionate mortality of individuals aged 80+ years due to COVID-19 relative to deaths at all ages due to COVID-19 by region and epidemiological week. Brazil, January to April 2021.**

**Supplementary figure 2. Proportionate mortality due to COVID-19 of individuals aged 70-79 and 80+ years relative to deaths at all ages due to COVID-19 by month. Brazil, May 2020 to May 2021.**

**Supplementary figure 3. Sex-specific proportionate mortality due to COVID-19 of individuals aged 70-79 and 80+ years relative to deaths at all ages due to COVID-19 by month. Brazil, May 2020 to May 2021.**
